# Prevalence and Genetic Characterizations of *Cryptosporidium* spp. in Pre-Weaned and Post-Weaned Piglets in Heilongjiang Province, China

**DOI:** 10.1371/journal.pone.0067564

**Published:** 2013-07-03

**Authors:** Weizhe Zhang, Fengkun Yang, Aiqin Liu, Rongjun Wang, Longxian Zhang, Yujuan Shen, Jianping Cao, Hong Ling

**Affiliations:** 1 Department of Parasitology, Harbin Medical University, Harbin, Heilongjiang, China; 2 College of Animal Science and Veterinary Medicine, Henan Agricultural University Zhengzhou, Henan, China; 3 National Institute of Parasitic Diseases, Chinese Center for Disease Control and Prevention, Key Laboratory of Parasite and Vector Biology, Ministry of Health, World Health Organization Collaborating Centre for Malaria, Schistosomiasis and Filariasis, Shanghai, China; Technion-Israel Institute of Technology Haifa 32000 Israel, Israel

## Abstract

**Background:**

*Cryptosporidium* spp. are common intestinal protozoa of humans and animals. There have been few studies conducted on the molecular characterizations of pig-derived *Cryptosporidium* isolates worldwide, especially in China. Thus, the aim of the present study was to understand the prevalence, distribution and genotypes of *Cryptosporidium* in pigs in Heilongjiang Province, China.

**Methodology/Principal Findings:**

A total of 568 fecal samples from pre-weaned and post-weaned piglets were collected from eight pig farms from four areas of Heilongjiang Province. The average infection rate of *Cryptosporidium* was 1.6% (9/568) by microscopy. 113 samples were subjected to PCR amplification of the small subunit (SSU) rRNA gene of *Cryptosporidium*, with 55.8% (63/113) being positive for *Cryptosporidium*. *Cryptosporidium suis* (n = 31) and *C. scrofarumn* (n = 32) were identified by DNA sequencing of the SSU rRNA gene. Three types of *C. scrofarumn* were found at the SSU rRNA locus, with one novel type being detected. Using species/genotype-specific primers for pig-adapted *Cryptosporidium* spp., 22 and 23 respectively belonged to *C. suis* and *C. scrofarum* mono-infections, with 18 co-infections detected. The infection peaks for *C. suis* (60%, 24/40) and *C. scrofarum* (51.2%, 21/41) were respectively found in the piglets of 5 to 8 weeks and more than 8 weeks.

**Conclusion/Significance:**

The detection of *C. suis* and *C. scrofarum* in pre-weaned and post-weaned piglets has public health implications, due to the fact that the two species are both zoonotic *Cryptosporidium*. The novel *C. scrofarum* type detected may be endemic to China.

## Introduction

*Cryptosporidium* spp. are common intestinal protozoa occurring in humans and many animal species, and do harm to the health of hosts. Animals and humans infected with *Cryptosporidium* are both potential sources of *Cryptosporidium* contamination in the environment. However, the contribution of each source is not clear, especially in developing countries. The distribution of *Cryptosporidium* spp. in humans and the human cryptosporidiosis burden of animal origin differ in different geographic regions. It has been proved that the transmission of *C. parvum* in humans is mostly anthroponotic in developing countries, with zoonotic infections play an important role in developed countries [1]. *Cryptosporidium* spp. are highly prevalent in livestock. Cattle have always been the subject of most of the studies in assessing the zoonotic potential of *Cryptosporidium* infections in animals. Years of epidemiological data have also documented the occurrence of natural infection of *Cryptosporidium* in other livestock. *Cryptosporidium* spp. have also been reported in pigs worldwide [2].

There is an extensive genetic variation within the genus *Cryptosporidium*, with 24 *Cryptosporidium* species having been recognized and more than 70 genotypes having been found [1], [3], [4]. To date, six *Cryptosporidium* species have been isolated from pigs, including *C. suis*, *C. scrofarum* (previously named as *Cryptosporidium* pig genotype II), *C. parvum*, *C. muris,* C. *tyzzeri* (previously named as *Cryptosporidium* mouse genotype I) and *C. andersoni* [2] (Table 1). Meanwhile, experimental infection studies revealed the susceptibility of pigs to *C. parvum*, *C. hominis* and *C. meleagridis* [5]–[10]. However, there are differences in the population structure and the molecular characterizations of *Cryptosporidium* species/genotypes in pigs between and within countries. Despite the fact that cryptosporidiosis in pigs does not always result in clinical signs, human cases infected with *C. suis* and *C. scrofarum* suggest that the two pig-adapted *Cryptosporidium* species are potentially zoonotic [11]–[14].

**Table 1:** *Cryptosporidium* species/genotypes of natural infection identified in pigs in different countries.

Country	No. of isolates	No.of *Cryptosporidium* species/genotypes (%)						Ref
		*C. suis*	*C. scrofarum*	*C. parvum*	*C. muris*	C. *tyzzeri*	*C. andersoni*	
Australia	95	43 (45.26)	50 (52.63)	2 (2.11)				[28], [33], [47], [51]–[54]
Brazil	2		2 (100.00)					[37]
Canada	179	52 (29.05)	90 (50.28)	33 (18.44)	3 (1.68)	1 (0.56)		[15], [29], [45]
China	202	157 (77.72)	42 (20.79)			2 (0.99)	1 (0.50)	[2], [16]–[19]
Czech Republic	694	495 (71.33)	195 (28.10)	2 (0.29)	2 (0.29)			[22], [24], [40], [42]
Denmark	184	51 (27.72)	133 (72.28)					[32], [43]
England	39	6 (15.38)	25 (64.10)	8 (20.51)				[26]
Ireland and N. Ireland	56	30 (53.57)	22 (39.29)	2 (3.57)	2 (3.57)			[25], [46]
Norway	9	6 (66.67)	3 (33.33)					[36]
Spain	26	10 (38.46)	16 (61.54)					[44]
Switzerland	6	2 (33.33)		4 (66.67)				[47], [51], [53]
Vietnam	14	12 (85.71)	2 (14.29)					[31]
Total	1506	864 (57.37)	580 (38.51)	51 (3.39)	7 (0.46)	3 (0.20)	1 (0.07)	

Pigs can be a source of infection for humans and other animals through direct or indirect contact with pig manure. The practice of using untreated pig manure (that may be positive for *Cryptosporidium* oocysts) directly as fertilizer on open crop or tillage land can cause environmental contamination. After a heavy rainfall, a large number of oocysts in animal slurry can enter streams and rivers from pasture run-off. Exposure to infective oocysts through contaminated water and food is an important route of transmission to humans [15].

In China, prevalence data have certificated cryptosporidiosis in pigs in various areas, with the average prevalence ranging from 8.9% to 56.8% [2]. However, molecular identification and genetic characterizations of *Cryptosporidium* spp. are only limited to small numbers of *Cryptosporidium* isolates [2], [16]–[19]. To date, no studies have been reported in pigs in Heilongjiang Province, China. The present study focused on the investigation of *Cryptosporidium* in pre-weaned and post-weaned piglets in Heilongjiang Province. The aim was to understand the prevalence, distribution and genotypes of *Cryptosporidium* in pigs by DNA sequencing of secondary PCR products of the SSU rRNA gene. Molecular epidemiological data would help us to understand the transmission dynamics of cross-*Cryptosporidium* species/genotypes between humans and pigs, and to assess the cryptosporidiosis burden attributable to zoonotic transmission by aligning the obtained sequences in the present study with those derived from humans available from GenBank.

## Materials and Methods

### Ethics Statement

Before beginning work on this study, we contacted the farm owners and obtained their permission to have their animals involved. During sample collection, all animal work followed guidelines in accordance with the Regulations for the Administration of Affairs Concerning Experimental Animals, and was approved by the Animal Ethical Committee of Harbin Medical University.

### Fecal Specimen Collection and Examination

In a one-year study from October 2011 to October 2012, 568 fecal samples were collected from eight intensive pig farms in four areas of Heilongjiang Province (Harbin, Daqing, Qiqihar and Mudanjiang). Farms were selected only according to each owner’s willingness and accessibility of animals for sampling. Approximate 20 g fresh fecal sample for each animal was collected immediately after being defecated on the ground of the pen by using a sterile disposal latex glove, and then placed in a disposable plastic bag individually. All the animals were healthy at the time of sampling, with their ages ranging from 15 to 105 days. Samples were transferred to the laboratory and stored in refrigerators at 4°C.

Before microscopic examination, fecal samples, accounting for approximate 20% of the samples of each age group in each farm, were randomly selected and sieved, and then stored (approximate 10 g each) in 2.5% potassium dichromate at 4°C prior to being used in molecular identification. A total of 113 fecal samples were collected, with 16 being less than 5 weeks old, 43 being 5–8 weeks old and 54 being more than 8 weeks old. Meanwhile, all the 568 samples were processed for microscopic examination. Oocysts in the fecal samples from pre-weaned piglets (less than 5-week-old) were concentrated by formalin-ethyl acetate sedimentation method to remove the fats in the samples and were stained by modified fast-acid staining technique. Sugar floatation method was used to concentrate oocysts in fecal samples from post-weaned piglets (equal to or more than 5-week-old). All the processes were finished in the laboratory within 48 hours after collection.

### DNA Extraction

Potassium dichromate was washed off with distilled water by centrifugation at 1500 g for 10 minutes four times at room temperature. DNA extraction was performed on 113 stored fecal samples. Genomic DNA of *Cryptosporidium* was extracted from 200 mg of each fecal sample using a commercially available kit (QIAamp DNA Mini Stool Kit, Qiagen, Hilden, Germany) in accordance with the manufacturer-recommended procedures. Eluted DNA was kept frozen at −20°C in refrigerators until PCR amplification.

### Genotyping of *Cryptosporidium*

An approximate 830bp fragment of the SSU rRNA gene was amplified from all DNA preparations by a nested PCR using genus-specific primers of *Cryptosporidium* as previously described [20]. All the secondary PCR products positive for *Cryptosporidium* were sequenced and identified to *Cryptosporidium* species/genotypes. For the assessment of mixed infection and age-specificity of *C. suis* and *C. scrofarum* in pigs, DNA preparations characterized as *C. suis* and *C. scrofarum* were respectively analyzed by a nested PCR protocol using pig-derived *Cryptosporidium-*specific primers to amplify a 443 bp fragment of the SSU rRNA gene from *C. scrofarum* and a 482 bp fragment of the SSU rRNA gene from *C. suis*, with the first set of genus-specific primers as described by Jiang et al and the second set of species/genotype-specific primers designed by Jenikova et al [21], [22]. All the secondary PCR products were sequenced to confirm if mixed *C. scrofarum* and *C. suis* infections were detected.

### DNA Sequence Analysis

All purified secondary PCR products were directly sequenced with secondary PCR primers on an ABI PRISMTM 3730 DNA Analyzer (Applied Biosystems, USA), using a BigDye Terminator v3.1 Cycle Sequencing kit (Applied Biosystems, USA). Accuracy of the sequencing data was confirmed by sequencing in both directions and additional PCR products if required. The SSU rRNA gene sequences obtained in the present study were aligned with each other and reference sequences obtained from GenBank by using Clustal X 1.83. Sequences of the partial SSU rRNA gene from representative isolates obtained were deposited in the GenBank database under accession numbers: KC481228 to KC481231.

## Results

### Prevalence and Age Distribution of *Cryptosporidium*

568 fecal samples were microscopically screened for the presence of *Cryptosporidium* oocysts. Meanwhile, 113 randomly selected fecal samples were screened for the presence of *Cryptosporidium* by PCR amplification of the partial SSU rRNA gene. *Cryptosporidium* oocysts were found in all the eight pig farms selected in the present study. In total, the average infection rate of *Cryptosporidium* was 1.6% (9/568) by microscopy versus 55.8% (63/113) by PCR. The highest infection rates were both found in 5–8 week-old pigs, with the prevalence 2.8% (6/216) by microscopy versus 83.7% (36/43) by PCR, followed by 1.1% (3/271) versus 42.6% (23/54) in more than 8-week-old pigs. In less than 5-week-old pigs, only four samples were positive for *Cryptosporidium* by PCR, whereas no *Cryptosporidium* oocysts were found by microscopy (Table 2). Infection intensity was observed to be low based on sporadic *Cryptosporidium* oocysts in smears of all the nine microscopy-positive fecal samples.

**Table 2:** Prevalence and age distribution of *Cryptosporidium* in pigs in Heilongjiang Province, China by microscopy and by PCR.

Age (week)	By microscopy		By PCR	
	No. of examined	No. of positive (%)	No. of examined	No. of positive (%)
<5	81	0	16	4 (25.0)
5–8	216	6 (2.8)	43	36 (83.7)
>8	271	3 (1.1)	54	23 (42.6)
Total	568	9 (1.6)	113	63 (55.6)

### Age-specific Distribution of *Cryptosporidium* Species/genotypes

A total of 63 fecal samples were successfully amplified at the SSU rRNA locus using the genus-specific nested PCR. DNA sequencing confirmed the presence of *C. suis* (n = 31) and *C. scrofarum* (n = 32) in piglets in the investigated areas. A combination of genus-specific and species/genotype-specific primers revealed 22 cases of *C. suis* mono-infection, 23 cases of *C. scrofarum* mono-infection and 18 cases of mixed infection of them both. Despite the two parasites appearing in all the age groups, *C. suis* (60%, 24/40) and *C. scrofarum* (51.2%, 21/41) had the highest percentage of positive samples in the pigs of 5 to 8 weeks and more than 8 weeks, respectively. *C. scrofarum* appeared to be more prevalent in older pigs than *C. suis* (Table 3).

**Table 3:** Age-specific distribution of *Cryptosporidium* species in pigs in Heilongjiang Province, China.

Age (week)	No. of positive	No. of *Cryptosporidium* spp.		
		*C. suis* mono-infection	*C. scrofarum* mono-infection	Mixed infection
<5	4	2	1	1
5–8	36	14	8	10
>8	23	6	14	7
Total	63	22	23	18

### Molecular Characterization of *Cryptosporidium* spp. at the SSU rRNA Locus

Sequence analysis of *Cryptosporidium* SSU rRNA gene revealed that all the 31 *C. suis* isolates were identical to each other (KC481228), having 100% similarity with the pig-derived sequences (JF710259, GQ227705 and GU254171-77 from China, AF108861 from Switzerland and EF489038 from Ireland). Three types were observed among 32 *C. scrofarum* isolates, with one to three nucleotide variations between one another. The most common type (KC481229) was found in 93.8% (30/32) of *C. scrofarum* samples and had 100% similarity with those isolates derived from a human (EU331243 from the Czech Republic), and pigs (HQ844734, GU254170, GQ227704 and GU254168 from China, DQ182600 from Denmark, GQ924105 from Brail, and JX424840 from Czech Republic). For the remaining two sequences, one (KC481231) had 100% similarity with a Chinese pig-derived sequence (HQ844730) and the other (KC481230) was never identical to any reported *C. scrofarum*.

## Discussion

Pigs have been reported to be infected naturally with *Cryptosporidium* worldwide. The prevalence of pig cryptosporidiosis varies between different countries and between different areas within a country. In the present study, the average infection rate of C*ryptosporidium* in pigs was 1.6% (9/568) by microscopy versus 55.8% (63/113) by PCR, with the highest infection rate being found in 5–8 week-old pigs by either of the two identification methods (2.8% versus 83.7%) (Table 2). Many factors are generally considered to influence infection rates of *Cryptosporidium* in various hosts, including age and health status of the infected hosts, the size and structure of samples (experimental design), the variety of detection methods employed and so on.

It is known that pigs of all ages are affected by *Cryptosporidium*. However, a number of studies on the relationship between the prevalence of *Cryptosporidium* and the age of pigs demonstrate that *Cryptosporidium* infections are the most common in piglets more than one month but generally less than six months of age [23]–[26]. In fact, infections are detected less frequently in piglets younger than one month or in adults, and even in some studies, there is an absence of *Cryptosporidium* oocysts [24], [27]. In the present study, *Cryptosporidium* were most frequently detected in 5–8 week-old piglets either by microscopy or by PCR (Table 2). The result was consistent with the previous reports that *Cryptosporidium* were mainly found in piglets within two months of weaning [2], [19], [22], [28]. Maddox-Hyttel et al and Farzan et al suggested that *Cryptosporidium* are more likely to be detected in post-weaned pigs than any other age group, and they also attribute the result to a reduction in immunity as animals lose the maternally immunity while their own immunity still needs to develop [15], [23]. A longitudinal study gave more exact data that piglets shed oocysts at the beginning of 45 days post weaning averagely [29]. However, two recent studies conducted in Vietnam have drawn the opposite conclusion that the prevalence of *Cryptosporidium* in pre-weaned pigs is significantly higher than that in post-weaned pigs [30], [31]. In another study of a pig cryptosporidiosis survey in eastern China’s Shanghai and Jiangsu, no age differences have been observed in the prevalence of *Cryptosporidium* [16]. To date, there has been no definitive conclusion about age distribution of *Cryptosporidium* in pigs. It is difficult to explain why conventionally reared piglets less than one month of age have been reliably infected under experimental conditions in previous studies [8], [9], [32].

In the present study, PCR was 34.9 times as sensitive as microscopy for the diagnosis of pig cryptosporidiosis, with the prevalence 55.8% (63/113) versus 1.6% (9/568) for PCR versus microscopy respectively. Not surprisingly, molecular techniques have a greater power of *Cryptosporidium* diagnosis. PCR-based detection was demonstrated to be more sensitive than microscopy in the two studies of the *Cryptosporidium* prevalence in sheep: in Australia, the prevalence was 2.6% by microscopy compared to 26.3% by PCR, and in the United States, *Cryptosporidium* was identified in 20.6% by microscopy compared to 50.8% by PCR [33], [34]. Although the prevalence of pig cryptosporidiosis conducted in Canada was 44.3% by microscopy versus 55.7% by PCR, there were statistically significant differences between the two testing methods [15]. The present higher ratio of PCR to microscopy might be attributable to the fact that all the animals were in a good health condition at the sampling and shed a lower number of oocysts in their feces. Thus, infection intensity of oocysts might be below the limit of detection of conventional morphological methods. Actually, pig fecal samples are generally reported to have lower intensity of oocysts than those from other animals based on recovery and enumeration of *Cryptosporidium* oocysts in their feces [16], [25], [31], [35], [36]. Therefore, PCR would be advised to be the preferred method for more accurate estimation of prevalence of *Cryptosporidium* in pigs. Besides the factors above, of course, differences in prevalence might be also related to differences in farm management systems. It has been reported that production in an intensive system might cause proliferation and maintenance of pathogens if techniques of handling are inadequate [37]. Therefore, measures should be taken to avoid the cross transmission of *Cryptosporidium* between different individuals within each farm.

DNA sequencing analysis of the partial SSU rRNA gene confirmed the presence of two *Cryptosporidium* spp. in 63 PCR-positive samples of *Cryptosporidium* in the present study, with 22 cases belonging to *C. suis* mono-infection, 23 cases belonging to *C. scrofarum* mono-infection and 18 cases belonging to mixed infection of them both. It has been reported previously that many hosts are susceptible to simultaneous infections with several *Cryptosporidium* spp., such as humans, cattle, pigs and so on [6], [16], [22], [38]–[40]. In fact, mixed infections in farm animals might be more prevalent than expected before. The identification of *Cryptosporidium* spp. is commonly based on DNA sequencing alone or in combination with PCR-RFLP analysis of the SSU rRNA gene fragment. However, the ability of these methods to identify mixed infection is limited compared to the use of species/genotype-specific PCR tools. This is because genus-specific primers will preferentially amplify the predominant species/genotypes due to the inherent nature of PCR [39], [41]. In the present study, the high percentage (28.6%, 18/63) of mixed infections may be due to the combined use of both the genus-specific and species/genotypes-specific primers for *C. suis* and *C. scrofarum* at the SSU rRNA locus. This may also explain why there are few reports of mixed infection cases in farm animals in previous studies as most previous studies did not use species/genotypes-specific primers.

The combination of genus-specific and species/genotypes-specific primers for *C. suis* and *C. srcofarum* not only enhanced our understanding of the population structure of C*ryptosporidium* in pigs but also helped us to clarify the age-related distribution of both parasites in pigs. In the present study, although *C. suis* and *C. scrofarum* were both found in all age groups, there appeared to be an age-related difference in the prevalence of pig cryptosporidiosis. The infection peaks for *C. suis* (60%, 24/40) and *C. scrofarum* (51.2%, 21/41) were respectively found in piglets that were 5 to 8 weeks old and more than 8 weeks old. Analysis of the current literature indicates that no clear conclusions can be drawn about the age distribution of *C. suis and C. scrofarum* in pigs. In general, *C. suis* seems to infect pigs of each age category, although the prevalence is lower in older pigs [3]. In a study of pig cryptosporidiosis in Czech Republic, *C. suis* was found to infect all 1–12–week-old pigs and showed the absence of age specificity of this species [22]. However, Kváč et al only identified *C. suis* in the pigs under 7 weeks of age [42]. It has been reported that *C. suis* preferentially infected sucking piglets [31], [43]. Conversely, *C. scrofarum* appears to be specific for older pigs compared to *C. suis* [22], [31], [40], [42]–[44], with a much lower prevalence in younger age categories, primarily in pre-weaned piglets [2], [43]. *C. scrofarum* was found only in pigs at the age of 2–6 months [44], with more common in weaned piglets [2], [28], [31]. There are more accurate data reported that *C. scrofarum* appeared in pigs older than 6 weeks in one study [22]. In another study, *C. scrofarum* isolates were only identified in pigs from 7 weeks of age and was not identified in pre-weaners [42]. However, in the present study, two *C. scrofarum* isolates were identified in pre-weaned pigs, with one being mono infection and the other being mixed infection with *C. suis*.

To date, six *Cryptosporidium* species/genotypes have been identified molecularly in pig fecal samples, with *C. suis* and *C. scrofarum* being the most common (Table 1). The findings of only *C. suis* and *C. scrofarum* in piglets in the present study supported the conclusion above. The absence of *C. parvum* infection in the investigated areas was in agreement with previous observation that pigs are not a major source of *C. parvum* [1], [42], [43]. However, there appeared to be geographical differences in the preference of *C. parvum* in pigs. No *C. parvum* has been found in pigs in China. To date, *C. parvum* has been identified in pigs in European countries as well as Australia and Canada (Table 1). Also, it is interesting that *C. parvum*, which generally tends to be infective for juvenile animals, has also been identified in mature sows and weaning piglets [25], [40], [45].

*C. muris*, C. *tyzzeri* and *C. andersoni* are minor *Cryptosporidium* species in pigs and were not been detected in the present study. To date, it is unclear whether the previous findings represented a natural infection of pigs although Chen and Huang suggested the possible transmission routes of C. *tyzzeri* between rodents and pigs [16]. It needs to be confirmed with more systematic studies of experimental infection of the three parasites, for we have no sufficient evidence to rule out the mechanical transmission of the oocysts in pig feces. *C. hominis* was previously reported in one pig fecal sample, however, it was considered to be a sequencing artifact [2], [25].

In the present study, DNA sequence analysis of the SSU rRNA gene indicated that all the 31 *C. suis* isolates were identical to each other, having 100% homology with the pig-derived sequences from Switzerland, Ireland and China [2], [25], [47]. Among the 32 *C. scrofarum* isolates, three types were found based on the SSU rRNA gene sequences, including one novel type. In general, *C. suis* was more conserved than *C. scrofarum*. To date, 10 types have been observed worldwide from 67 SSU rRNA nucleotide sequences available in GenBank, which represented 895 *C. suis* isolates (including 31 in the present study). However, among 612 *C. scrofarum* isolates (including 32 in the present study), 16 types have been found in 46 SSU rRNA nucleotide sequences available in GenBank. By aligning obtained sequences with those from GenBank, it has been noticed that the SSU rRNA nucleotide sequence of the most prevalent type of *C. scrofarum* (93.75%, 30/32) (KC481229) had 100% homology with that from a diarrheal stool of 29-year-old immune-competent man (EU331243) [11]. Thus, the pigs infected with *C. scrofarum* may pose a threat to local inhabitants and may be of public health significance. *C. suis,* which is considered to have the potential to be zoonotic pathogens, has also been isolated in humans [12]–[14]. Due to the only sporadic humans cases infected with *C. suis* and *C. scrofarum* reported worldwide, we had no sufficient data available to assess the burden of human cryptosporidiosis caused by the two parasite attributable to zoonotic transmission.

*C. suis* and *C. scrofarum* appear to be adapted to pigs. Pig cryptosporidiosis might be paid less attention than it is supposed to due to the fact that the pigs infected with two parasites are generally reported to be asymptomatic [1], [42], [43]. Thus, pigs might have more opportunity to continually shed human-infective oocysts of *C. suis* and *C. scrofarum* into the environment through their feces. In fact, oocysts of *C. suis* and *C. scrofarum* have been found in water environment in some areas in China, including source water for drinking water plant, wastewater nearby pig farms as well as raw domestic wastewater in a wastewater treatment plant [48]–[50]. It is well-known that oocysts are extremely easily spread via water and it is difficult to remove or eliminate the parasites in water supply. Due to the lack of data of human cryptosporidiosis in the investigated area, even in China, the true prevalence of human cryptosporidiosis caused by *C. suis* and *C. scrofarum*, the transmission dynamic and the disease burden attributable to the two parasites of pig origin need to be assessed by more extensive molecular epidemiological surveys from humans and animals in the future. Unique SSU rRNA gene sequences of *C. scrofarum* in pigs in the investigated areas may reflect characteristic geographical distribution. The present data will help local authorities to develop protective strategies for the prevention and control of cryptosporidiosis in Heilongjiang Province. Considering the high prevalence of both parasites in pigs, further studies also need to be focused on the relationship between pig cryptosporidiosis and different farm breeding systems. It is important to develop better farm management systems to prevent the occurrence of cross transmission and re-infection of *Cryptosporidium* among the animals within each farm, and to reduce environmental contamination by reducing zoonotic agents from pig manure.
